# Controlling antimicrobial resistance in hospitals: infection control and use of antibiotics.

**DOI:** 10.3201/eid0702.010206

**Published:** 2001

**Authors:** R A Weinstein

**Affiliations:** Cook County Hospital and Rush Medical College, Chicago, Illinois 60612, USA. rweinste@rush.edu

## Abstract

Antimicrobial-drug resistance in hospitals is driven by failures of hospital hygiene, selective pressures created by overuse of antibiotics, and mobile genetic elements that can encode bacterial resistance mechanisms. Attention to hand hygiene is constrained by the time it takes to wash hands and by the adverse effects of repeated handwashing on the skin. Alcohol-based hand rubs can overcome the time problem and actually improve skin condition. Universal glove use could close gaps left by incomplete adherence to hand hygiene. Various interventions have been described to improve antibiotic use. The most effective have been programs restricting use of antibiotics and computer-based order forms for health providers.


### Controlling Antimicrobial Resistance in Hospitals: Infection Control and Use of Antibiotics

**Robert A. Weinstein**
Cook County Hospital and Rush Medical College, Chicago, Illinois, USA

Antimicrobial-drug resistance in hospitals is driven by failures of hospital hygiene, selective pressures created by overuse of antibiotics, and mobile genetic elements that can encode bacterial resistance mechanisms. Attention to hand hygiene is constrained by the time it takes to wash hands and by the adverse effects of repeated handwashing on the skin. Alcohol-based hand rubs can overcome the time problem and actually improve skin condition. Universal glove use could close gaps left by incomplete adherence to hand hygiene. Various interventions have been described to improve antibiotic use. The most effective have been programs restricting use of antibiotics and computer-based order forms for health providers.

The forces that drive antimicrobial-drug resistance (failures of hospital hygiene, selective pressures created by overuse of antibiotics, and mobile genetic elements that can encode bacterial resistance mechanisms) have been discussed at length (1-4). Despite this extensive knowledge base, exhortations about resistance, and formal control guidelines (5), drug resistance has continued to emerge, especially in intensive care units (ICUs) (Figure 1).

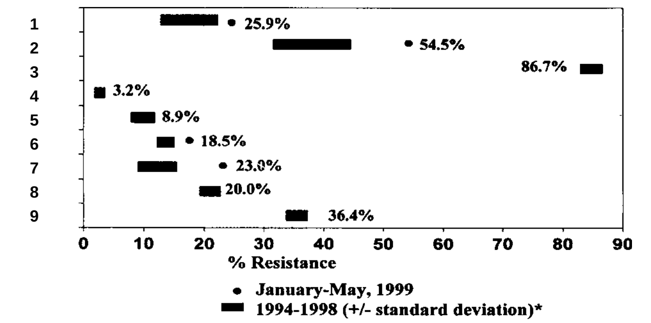

1 = Vancomycin/enterococci
2 = Methicillin/*S. aureus*
3 = Methicillin/CNS
4 = 3rd Ceph/*E. coli***
5 = 3rd Ceph/*K. pneumoniae***
6 = Imipenem/*P. aeruginosa*
7 = Quinolone/*P. aeruginosa*
8 = 3rd Ceph/*P. aeruginosa*
9 = 3rd Ceph/*Enterobacter* spp.

Note: *S. aureus* = *Staphylococcus aureus*; CNS = coagulase-negative staphylococci; 3rd Ceph = reistance to third-generation cephalosporins (ceftriaxone, cefotaxime, or ceftazidime); *P. aeruginosa* = *Pseudomonas aeruginosa*; Quinolone = resistant to either ciprofloxacin or ofloxacin.
*Percentage (%) increase in resistance rate of current period (January-December 1999) compared to mean rate of resistance over previous 5 years (1994 through 1998): [(1999 rate - previous 5-year mean rate)/previous 5-year mean rate] X 100.
**Resistance for *Escherichia coli* or *Klebsiella pneumonia* is the rate of nonsusceptibility of these organisms to either 3rd Ceph group or aztreonam.

Figure 1. Rates of resistance in nosocomial infections reported in ICU patients, National Nosocomial Infections Surveillance System, CDC. Comparison of data from January-December 1999 with historical data.

Address for correspondence: Robert A. Weinstein, Division of Infectious Diseases – Suite 129 Durand, Cook County Hospital, 1835 W. Harrison St.,Chicago, IL 60612, USA; fax: 312-572-3523; e-mail: rweinste@rush.edu

In a survey in four U.S. medical centers (a public hospital, a community hospital, a long-term care facility, and a university hospital), 85% of 424 physicians noted that antimicrobial-drug resistance was a major national problem; 55% thought that resistance was an issue for their patients (6). At the root of the resistance problem are health-care workers, who, although generally willing to do the right thing to control antimicrobial-drug resistance, undervalue the problem, do not know what the "right thing" is, or need an easier way to do it. This review summarizes a "facilitated right thing" approach to the problems of failed hygiene and antibiotic pressures.

### Hand Hygiene

In a recent survey of physicians (6), 45% considered poor handwashing practices an important cause of antimicrobial-drug resistance in hospitals, perhaps a reflection of health-care workers' markedly inflated view of their attention to hand hygiene (Table 1) (7). In fact, in most surveys of handwashing adherence, in various patient-care settings, personnel have practiced appropriate hand hygiene in only 25% to 50% of opportunities. As we pass the sesquicentennial of Semmelweis' seminal observations on the importance of hand hygiene in reducing the incidence of nosocomial childbed fever, why does handwashing remain the most breached infection control measure in hospitals? Two frequently cited reasons are the large time commitment (up to

Table 1. Hospital personnel self-reported and observed handwashing rates[a]

| | Handwashing after patient contact N (%) |
|---|---|
| Self-reported rate (n=123) | 104 (85) |
| Estimate of co-workers' rate (n=123) | 63 (51) |
| Observed rate (n=173) | 48 (28) |

[a]From Chicago Antimicrobial Resistance Project and from data adapted from Vernon et al. (7).

90 minutes per work shift if performed as recommended by the Centers for Disease Control and Prevention [CDC]) and the adverse effects of repeated handwashing on the skin (8).

### Alcohol-Based Hand Rubs

If given a choice of changing human behavior (e.g., improving attention to hygiene and asepsis) or designing a technologically foolproof device to control infections, go for the device. For hand hygiene, we have the opportunity to fulfill the infection control "prime directive": use technologic advances to improve behavior. How? Alcohol-based sinkless hand rubs (Table 2) can overcome the time problems of handwashing (9) and actually improve skin condition (10). Handwashing requires approximately 45 to 90 seconds to access and use a sink with running water, soap, and hand-drying facilities; an alcohol-based hand rub can degerm hands in less than 30 seconds and enhance killing of transient hand flora.

Although use of alcohol for handwashing or scrubbing is perceived as leading to dry skin, use of alcohol hand rubs, without rinsing, is beneficial to skin, presumably because the protective fats and oils remain on the hands as the alcohol dries and because alcohol rubs contain emollients. In a study comparing an alcohol gel hand rub to soap and water handwashing, Boyce et al. reported that health-care workers found that alcohol hand rub causes less skin dryness, is accessible and convenient to use, and has a pleasant odor. After the study, 92% of test participants agreed to use the hand rub routinely (11).

Table 2. Potential benefits of alcohol-based sinkless hand degerming agents

| | Soap and water handwashing | Alcohol hand rub |
|---|---|---|
| Time required | 30-120 seconds | 10-30 seconds |
| Efficacy in degerming | Good to very good | Excellent |
| Acceptance by personnel | Historically poor | Good to excellent |

### Colonization Pressure and Universal Glove use

While alcohol-based hand rubs appear promising, maintaining adherence may require ongoing educational reenforcement, compliance monitoring, and feedback to personnel. With such aggressive campaigns, hand hygiene rates of 60% to 80% can be achieved. But is this enough? For uncommon pathogens that may colonize or infect only a small proportion of patients, indirect patient-to-patient cross-transmission by the hands of health-care workers may be interrupted readily by such adherence rates. However, when "colonization pressure" is greater because of a large number of colonized patients, such rates may not be sufficient. For example, when 30% to 50% of patients are colonized with vancomycin-resistant enterococci (VRE), even occasional lapses in hand hygiene may be enough to sustain cross-transmission (Figure 2) (12,13).

A "belt and suspenders" approach to the colonization pressure dilemma has been to encourage use of disposable examination gloves during contacts with patients and their environment (2,14,15). In one study, the rate of nosocomial

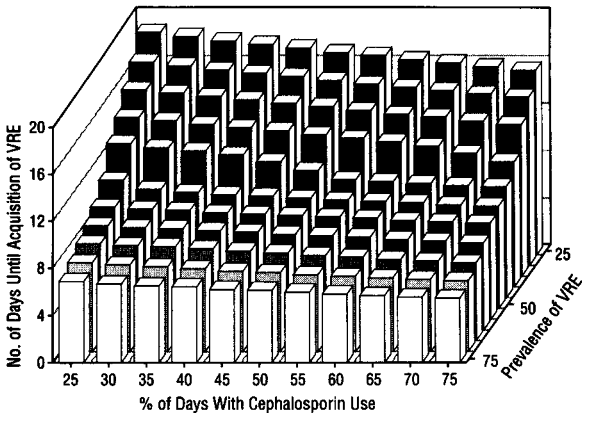

Figure 2. Median number of days until acquisition of VRE in a medical ICU; prevalence of VRE ("colonization pressure") exerted a greater effect on acquisition than did antibiotic use, i.e., time to acquisition of VRE was shorter with high colonization pressure and low antibiotic use than with the converse conditions (13).

*Clostridium difficile*-associated diarrhea was threefold lower on "universal glove use" wards than on control wards (16). In a study of VRE, 39% of personnel had contamination of examination gloves by VRE after even brief contact with infected or colonized patients; personnel hand contamination was reduced 71% by use of gloves (17). Because even intact upper body skin may be colonized by resistant bacteria such as VRE (18) and environmental contamination by VRE is common (19), we recommend that disposable examination gloves be worn for all contact, even with intact skin or the environment of at-risk patients. Gloves must be changed and hands disinfected by an alcohol hand rub between patients, because gloves are not a total barrier (17,20). In one observational study of universal glove use, 96% of gloved personnel removed gloves after leaving the patient's room (21). In that study, personnel cited a marked preference for universal glove use over traditional contact precautions.

Because of the huge resistance iceberg (Figure 3), with as many as 5 to 10 patients colonized with resistant bacteria for every patient known to be infected, universal glove use may be a more preferable infection control strategy than contact precautions, which are applied only to the tip of the iceberg. With universal glove use, gowning of personnel is recommended only for self-protection, e.g., from blood and body fluid exposures. In a study of the epidemiology and control of VRE in a medical ICU and in a study of control of VRE, methicillin-resistant *Staphylococcus aureus,* and ceftazidime-resistant *Escherichia coli* and *Klebsiella pneumoniae*, gowns did not add value to universal glove use (21,22). However, gowns may be of value for motivation (they have increased compliance in some studies) (22), in outbreak control (23), or in some heavily contaminated environments such as burn units.

### Prescription of Antibiotics

Antibiotic pressures may be more amenable to intervention than hygiene practices. Prescribers want to do

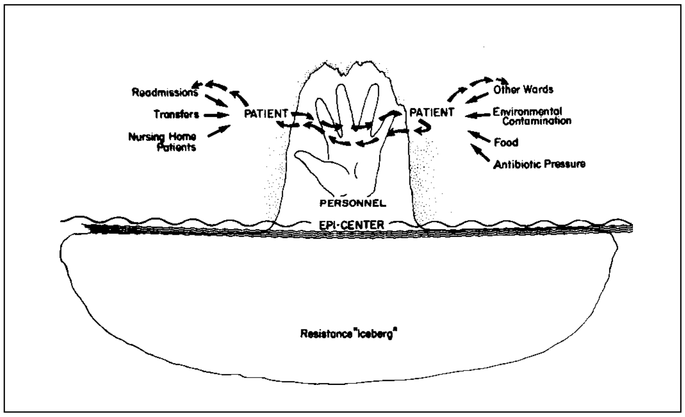

Figure 3. The dynamics of nosocomial resistance. Resistance iceberg floating in an epicenter (2).

the right thing but may not always remember recommendations. Even though most health-care workers see inappropriate use of antibiotics as an important cause of drug resistance, many consider use of broader-spectrum antibiotics for longer periods the way to stamp out resistant bacteria (6).

To simplify prescription of antibiotics, most hospitals use “closed” formularies that limit prescribing options, often based on competitive bidding, to one or two drugs per antibiotic class. Clinical guidelines have become popular, especially for common infections, such as community-acquired pneumonia. Such guidelines may improve antibiotic use, especially if results are audited, and feedback is provided to prescribers. Use of order forms (24) and concurrent feedback to prescribers or next-day review of antibiotic appropriateness (25) also can improve prescriptions. The most effective antibiotic interventions have been restriction programs and computer-based order forms (so-called provider-order entries).

### Restrictions to Use of Antibiotics

Restricting use of antibiotics has been especially effective in reducing cost and excess empiric use of broad-spectrum drugs (26). In one large study of the effect of prior authorization for selected drugs, a 32% decrease in expenditure for parenteral antibiotics was accompanied by increased susceptibility of bacterial isolates to beta-lactam and quinolone antibiotics. There were no adverse effects on clinical outcomes as measured by time to receipt of appropriate antibiotics, survival, and discharge from hospital for patients with bacteremia caused by gram-negative bacilli (27).

### Computer Order Entry

Computer-based order entry for medical providers uses technology to direct and improve prescription behavior and thus fulfills the infection control prime directive (28). Order entry systems for antibiotics (and other drugs) provide simple messages to prescribers, such as the hospital’s suggested indications for, or the local resistance patterns of, a selected antibiotic. More sophisticated systems integrate results of microbiology and other laboratory tests into decision-support algorithms (29). Because they provide prescribing information when it is needed, in a neutral, nonjudgmental, fact-based format, computer order forms are efficient and well accepted and can change prescribing behavior dramatically, almost overnight.

### Rotating Use of Antibiotics

The most recent intervention in antibiotic prescribing has been renewed interest in rotating use, or cycling, of antibiotics (30). Over 20 years ago, in a series of studies at the Minneapolis Veterans’ Administration Hospital, the substitution of amikacin for gentamicin and tobramycin as the aminoglycoside of choice produced sustained decreases in the prevalence of aminoglycoside-resistant gram-negative bacilli (31). The higher serum levels of amikacin, and the infrequent appearance in U.S. hospitals of amikacin-modifying enzymes that could confer amikacin resistance in gram-negative bacilli, were the underpinnings of the success of this strategy.

The more recent reports on cycling describe replacement (or switch) therapy for empiric antibiotic choices (30,32-34). Replacing ceftazidime with ciprofloxacin for empiric treatment of suspected gram-negative bacterial infections in a cardiac surgery ICU was associated with decreased incidence of ventilator-associated pneumonia and bacteremia caused by antibiotic-resistant gram-negative bacilli (33). In another hospital, use of beta-lactam/beta-lactamase inhibitor combinations to replace use of third-generation cephalosporins and clindamycin was associated with decreased rates of colonization by VRE (34); a follow-up study reported that these formulary manipulations were associated with decreasing numbers of patients from whom methicillin-resistant *S. aureus* and ceftazidime-resistant *K. pneumoniae* were cultured but increased rates of resistant *Acinetobacter* (35). Rotating use of fourth-generation cephalosporins, quinolones, carbapenems, and beta-lactam/beta-lactamase inhibitor combinations is being studied in several hospital ICUs.

Cycling of antibiotics is most likely to be effective for limited periods in closed environments, such as ICUs, but this approach requires careful microbiologic monitoring because of the monotonic selective pressure of a single agent and the possible emergence of resistance to unrelated classes of drugs caused by genetic linkage of resistance mechanisms (30,36). As the size of the patient population under study increases, availability of various classes of drugs may be more effective at reducing the risk of emergence of resistance and may be a better strategy than cycling (37).

### Conclusions

Control of antibiotic resistance requires aggressive implementation of several strategies (2): ongoing surveillance of resistance; molecular typing of isolates, usually using pulsed-field gel electrophoresis (38,39) when rates of resistance increase; using hygiene controls to limit spread of single (clonal) strains and antibiotic controls to limit spread of multiple (polyclonal) strains of resistant bacteria; and enlisting administrative support. Monitoring adherence of health-care workers to control measures and feedback of individual and ward rates of hygiene adherence and antibiotic resistance are central components of health-care worker education and motivation. Mathematical modeling has been used to judge the value of infection control activities. In these calculations, screening and cohorting of infected and colonized patients are the most effective control measures (11), although creating and maintaining cohorts are often logistically and technically difficult.

Current infection control strategies are aimed at the hygiene and antimicrobial engines that drive resistance. To ulfill the infection control prime directive, we must harness technology to improve and direct adherence to these strategies. Future approaches may control or eliminate the bacterial events that underlie evolution of resistance.

Dr. Weinstein is chair, Division of Infectious Diseases, Cook County Hospital; director of Infectious Disease Services for the Cook County Bureau of Health Services; and professor of medicine, Rush Medical College. He also oversees the CORE Center for the Prevention, Care and Research of Infectious Disease and directs the Cook County Hospital component of the Rush/Cook County Infectious Disease Fellowship Program. His areas of research include nosocomial infections (particularly the epidemiology and control of antimicrobial resistance and infections in intensive care units) and health-care outcomes for patients with HIV/AIDS.

### References

1. Sherris JC. The epidemiology of drug resistance. In: Proceedings of the International Conference on Nosocomial Infections. Atlanta: Center for Disease Control; 1970. p. 50-60.
2. Weinstein RA, Kabins SA. Strategies for prevention and control of multiple-drug resistant nosocomial infections. Am J Med 1981;70:449-54.
3. Tenover FC. Novel and emerging mechanisms of antimicrobial resistance in nosocomial pathogens. Am J Med 1991;91:76S-81S.
4. Weinstein RA. Epidemiology and control of nosocomial infections in adult intensive care units. Am J Med 1991;91:179S-184S.
5. Goldmann DA, Weinstein RA, Wenzel RP, Tablan OC, Duma RJ, Gaynes RP, et al. Strategies to prevent and control the emergence and spread of antimicrobial-resistant microorganisms in hospitals. A challenge to hospital leadership. JAMA 1996;275:234-40.
6. Wester CW, Durairaj L, Schwartz D, Husain S, Martinez E, Evans AT. Antibiotic resistance: who cares? Physician perceptions of antibiotic resistance among inpatients: its magnitude, causes, and potential solutions [abstract #529]. In: Proceedings of the 37th Annual Meeting of the Infectious Diseases Society of America, 1999 Nov 18-21, Philadelphia. Alexandria (VA): Infectious Diseases Society of America; 1999.
7. Vernon MO, Trick WB, Schwartz D, Welbel SF, Wisniewski M, Fornek ML, et al. Marked variation in perceptions of antimicrobial resistance (AR) and infection control (IC) practices among healthcare workers (HCWs). In: Proceedings of APIC 2000, June 18-22, Minneapolis, MN. St. Louis: Mosby, Inc.; 2000.
8. Larson E. Skin hygiene and infection prevention: more of the same or different approaches? Clin Infect Dis 1999;29:1287-94.
9. Pittet D, Mourouga P, Perneger TV. Compliance with handwashing in a teaching hospital. Ann Intern Med 1999;130:126-30.
10. Boyce J. Antiseptic technology: access, affordability, and acceptance. Emerg Infect Diseases 2001;7(2). In press.
11. Boyce JM, Kelliher S, Vallande N, Korber S, Denicola G, Fedo J. Hand disinfection with an alcoholic gel causes less skin irritation and dryness of nurses' hands than soap and water handwashing.[abstract #78]. In: Proceedings of the 9th Annual Society for Healthcare Epidemiology of America Meeting, April 18-20, 1999, San Francisco, CA. Thorofare (NJ): Slack Inc.; 1999.
12. Austin DJ, Bonten MJM, Weinstein RA, Slaughter S, Anderson RM. Vancomycin-resistant enterococci in intensive-care hospital settings: transmission dynamics, persistence and the impact of infection control programs. Proc Natl Acad Sci U S A 1999;96:6908-13.
13. Bonten JM, Slaughter S, Ambergen A, Hayden MK, Van Voorhis J, Nathan C, et al. The role of "colonization pressure" in the spread of vancomycin-resistant enterococci. Arch Intern Med 1998;158:1127-32.
14. Weinstein RA, Nathan C, Gruensfelder R, Kabins SA. Endemic aminoglycoside resistance in gram-negative bacilli. Epidemiology and mechanisms. J Infect Dis 1980;141:338-45.
15. Weinstein RA, Hayden MK. Multiply drug-resistant pathogens: epidemiology & control. In: Bennett JV, Brachman PS, editors. Hospital infections. 4th ed. Philadelphia: Lippincott-Raven; 1998. p. 215-36.
16. Johnson S, Gerding DN, Olson MN, Weiler MD, Hughes RA, Clabots CR, et al. Prospective, controlled study of vinyl glove use to interrupt *Clostridium difficile* nosocomial transmission. Am J Med 1990;88:137-40.
17. Badri SM, Sahgal NB, Tenorio AR, Law K, Hota B, Matushek M, et al. Effectiveness of gloves in preventing the transmission of vancomycin-resistant *Enterococcus* (VRE) during patient care activities. In: Program of the 36th Annual Meeting of the Infectious Diseases Society of America, November 11-14, 1998, Denver, CO, Abstract #599.
18. Beezhold D, Slaughter S, Hayden MK, Matushek M, Nathan C, Trenholme GM, et al. Skin colonization with vancomycin-resistant enterococci among hospitalized patients with bacteremia. Clin Infect Dis 1997;24:704-6.
19. Bonten MJM, Hayden MK, Nathan C, Van Voorhis J, Matushek M, Slaughter S, et al. Epidemiology of colonization of patients and environment with vancomycin-resistant enterococci. Lancet 1996;348:1615-19.
20. Olsen RJ, Lynch P, Coyle MB, Cummings J, Bokete T, Stamm WE. Examination gloves as barriers to hand contamination in clinical practice. JAMA 1993;270:350-3.
21. Trick WE, DeMarais PL, Jarvis WR, Tomaska W, Ohlrich S, Hageman J, et al. Comparison of universal gloving to contact isolation precautions to prevent transmission of multidrug-resistant bacteria in a long-term care facility. In: Proceedings of the 4th Decennial International Conference, Atlanta, Georgia, March 5-9, 2000. Thorofare (NJ): Slack, Inc.; 2000.
22. Slaughter S, Hayden MK, Nathan C, Hu TC, Rice T, Van Voorhis J, et al. A comparison of the effect of universal use of gloves and gowns with that of glove use alone on acquisition of vancomycin-resistant enterococci in a medi-cal intensive care unit. Ann Intern Med 1996;125:448-56.
23. Boyce JM, Opal SM, Chow JW, Zervos MJ, Potter-Bynoe G, Sherman CB, et al. Outbreak of multidrug-resistant *Enterococcus faecium* with transferable vanB class vancomycin resistance. J Clin Microbiol 1994;32:1148-53.
24. Durbin WA, Lapidas B, Goldmann DA. Improved antibiotic usage following introduction of a novel prescription system. JAMA 1981;246:1796-800.
25. Kortas K, Segreti J, Donnelly A, Pierpaoli P, Trenholme G, Levin S. An anti-infective review and monitoring program. Pharmacol Ther 1993;291-6.
26. Woodward RS, Medoff G, Smith MD, Gray JL. Antibiotic cost savings from formulary restrictions and physician monitoring in a medical-school-affiliated hospital. Am J Med 1987;83:817-23.
27. White AC, Atmar RL, Wilson J, Cate TR, Stager CE, Greenberg SB. Effects of requiring prior authorization for selected antimicrobials: expenditures, susceptibilities, and clinical outcomes. Clin Infect Dis 1997;25:230-9.
28. Schiff GD, Rucker TD. Computerized prescribing: building the electronic infrastructure for better medication usage. JAMA 1998;279:1024-9.
29. Evans RS, Pestotnik SL, Classen DC, Clemmer TP, Weaver LK, Orme JF, et al. A computer-assisted management program for antibiotics and other anti-infective agents. N Engl J Med 1998;338:232-8.

30. John JF. Antibiotic cycling: is it ready for prime time? Infect Control Hosp Epidemiol 2000;21:9-11.
31. Gerding DN. Antimicrobial cycling: lessons learned from the aminoglycoside experience. Infect Control Hosp Epidemiol 2000;21:S12-S17.
32. Rahal JJ, Urban C, Horn D, Freeman K, Segal-Maurer S, Maurer J, et al. Class restriction of cephalosporin use to control total cephalosporin resistance in nosocomial *Klebsiella*. JAMA 1998;280:1233-7.
33. Kollef MH, Vlasnik J, Sharpless L, Pasque C, Murphy D, Fraser V. Scheduled change of antibiotic classes: a strategy to decrease the incidence of ventilator-associated pneumonia. Am J Respir Crit Care Med 1997;156:1040-8.
34. Quale J, Landman D, Saurina G, Atwood E, DiTore V, Patel K. Manipulation of a hospital antimicrobial formulary to control an outbreak of vancomycin-resistant enterococci. Clin Infect Dis 1996;23:1020-5.
35. Landman D, Chockalingam M, Quale JM. Reduction in the incidence of methicillin-resistant *Staphylococcus aureus* and ceftazidime-resistant *Klebsiella pneumoniae* following changes in a hospital antibiotic formulary. Clin Infect Dis 1999;28:1062-6.
36. Wiener J, Quinn JP, Bradford PA, Goering RV, Nathan C, Bush K, et al. Multiple antibiotic-resistant *Klebsiella* and *Escherichia coli* in nursing homes. JAMA 1999;281:517-23.
37. Bonhoeffer S, Lipsitch M, Levin BR. Evaluating treatment protocols to prevent antibiotic resistance. Proc Natl Acad Sci U S A 1997;94:12106-11.
38. Tenover FC, Arbeit RD, Goering RV, Murray BE, Persing DH, Pfaller MA, et al. How to select and interpret molecular strain typing methods for epidemiological studies of bacterial infections: A review for healthcare epidemiologists. Infect Control Hosp Epidemiol 1997;18:426-39.
39. Matushek MG, Bonten MJ, Hayden MK. Rapid preparation of bacterial DNA for pulsed-field gel electrophoresis. J Clin Microbiol 1996;34:2598-600.
